# Using DNA Metabarcoding to Characterize the Prey Spectrum of Two Co-Occurring *Themisto* Amphipods in the Rapidly Changing Atlantic-Arctic Gateway Fram Strait

**DOI:** 10.3390/genes13112035

**Published:** 2022-11-04

**Authors:** Annkathrin Dischereit, Owen S. Wangensteen, Kim Præbel, Holger Auel, Charlotte Havermans

**Affiliations:** 1 Helmholtz Young Investigator Group ARJEL, Functional Ecology, Alfred Wegener Institute, Helmholtz Centre for Polar and Marine Research, 27570 Bremerhaven, Germany; 2 BreMarE—Bremen Marine Ecology, FB2, Universität Bremen, 28334 Bremen, Germany; 3 Faculty for Biosciences, Fisheries and Economics, Norwegian College of Fishery Science, UiT the Arctic University of Norway, 9019 Tromsø, Norway; 4 Department of Evolutionary Biology, Ecology and Environmental Sciences and Biodiversity Research Institute (IRBIO), University of Barcelona, 08007 Barcelona, Spain

**Keywords:** diet analyses, Hyperiidae, *Themisto libellula*, *Themisto abyssorum*, Arctic Ocean, atlantification, cytochrome c oxidase subunit I, DNA metabarcoding, gelatinous zooplankton

## Abstract

The two congeneric hyperiids *Themisto libellula* and *T. abyssorum* provide an important trophic link between lower and higher trophic levels in the rapidly changing Arctic marine ecosystem. These amphipods are characterized by distinct hydrographic affinities and are hence anticipated to be impacted differently by environmental changes, with major consequences for the Arctic food web. In this study, we applied DNA metabarcoding to the stomach contents of these *Themisto* species, to comprehensively reveal their prey spectra at an unprecedented-high-taxonomic-resolution and assess the regional variation in their diet across the Fram Strait. Both species feed on a wide variety of prey but their diet strongly differed in the investigated summer season, showing overlap for only a few prey taxa, such as calanoid copepods. The spatially structured prey field of *T. libellula* clearly differentiated it from *T. abyssorum,* of which the diet was mainly dominated by chaetognaths. Our approach also allowed the detection of previously overlooked prey in the diet of *T. libellula*, such as fish species and gelatinous zooplankton. We discuss the reasons for the differences in prey spectra and which consequences these may have in the light of ongoing environmental changes.

## 1. Introduction

The Arctic is changing at a faster pace than elsewhere on Earth. During the last decades, sea-ice extent and thickness have dramatically decreased [1,2]. Some models even predict a sea-ice free Arctic in the summer months to happen within one or two decades [3]. With the loss of multi-year sea-ice, an important and unique habitat for ice algae will disappear [4,5,6]. Ice-algal production is an important food source for many primary consumers [7,8,9,10]. As an example, the dominant Arctic copepod *Calanus glacialis* depends on the sea-ice algal bloom for maturation and reproduction, whereas the pelagic phytoplankton bloom later in the season is crucial for feeding its offspring [11,12]. As these grazers represent an important link between the sympagic production and higher trophic levels [13], sea-ice melting will have wide-ranging consequences for the entire Arctic ecosystem.

**Figure 1:**
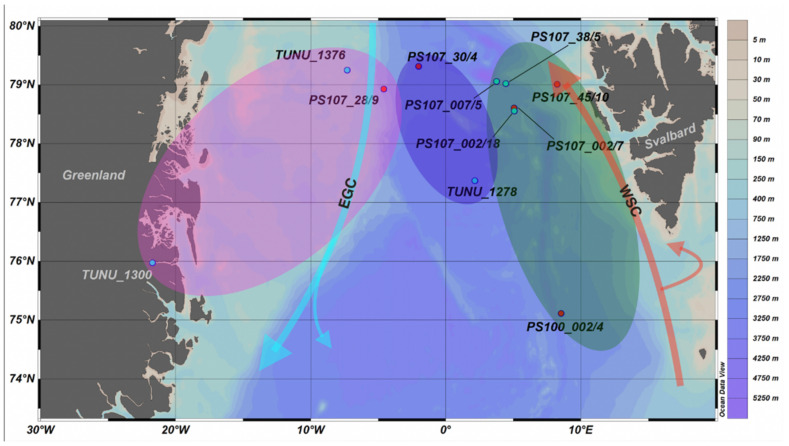
Sampling stations of *T. libellula* (blue) and *T. abyssorum* (red) in Fram Strait during two R/V Polarstern cruises (PS100; PS107) and a R/V Helmer Hansen cruise (TUNU) in 2016 and 2017. Circles indicate regions used for statistics assigned by geographical location: EGC (East Greenland Current and shelf) (pink), CFS (Central Fram Strait) (blue), WSC (West Spitsbergen Current) (green). Arrows indicate major currents in the Fram Strait.

The Fram Strait, located between Greenland and Svalbard is the only deep connection between the Arctic and Atlantic Oceans and it is influenced by two opposing currents [14,15] (Figure 1). In the east, along the Svalbard slope, the West Spitsbergen Current (WSC) brings warm Atlantic waters into the Arctic [15]. In the west, the East Greenland Current (EGC) transports cold Arctic water and sea-ice southwards along the east coast of Greenland [15]. Through a phenomenon called the “Atlantification” of the Arctic Ocean, the inflow of incoming warm Atlantic waters has recently increased, and this will further cause sea-ice decline in the eastern Eurasian Basin [16]. The WSC not only carries heat into the Arctic, but also boreal and sub-Arctic species, that might replace the cold-adapted and/or sea-ice dependent polar species and further impact the Arctic ecosystem and its food web structure [17,18,19].

In the Arctic Ocean, two hyperiid amphipod species are important components of the zooplankton community, the polar *Themisto libellula* and the boreal-Atlantic *T. abyssorum* [20,21,22]. These two congeneric species, co-existing throughout the Arctic region, are an important food source for higher trophic levels, including fish, seabirds, harp seals and whales [23]. They are themselves efficient predators of zooplankton, believed to mainly prey on calanoid copepods [15,24]. Hence, *Themisto* amphipods build an important link between lower and higher trophic levels in the Arctic marine food web. The ongoing rapid environmental changes in the Arctic, including the Atlantification, are also impacting *Themisto* amphipods. In recent years, it was observed that *T. abyssorum*, due to its affinity to Atlantic waters, became more abundant in the Barents Sea and Fram Strait, while *T. libellula*, dependent on colder, polar waters, has decreased in these regions [25]. Since higher trophic levels preferably feed on the larger *T. libellula*, a change in abundance and distribution of *T. libellula* and *T. abyssorum* could have severe consequences for the entire Arctic food web [23].

According to previous studies [8,24,26,27] *Themisto* amphipods are believed to be opportunistic carnivores feeding on the most abundant zooplankton species, including copepods, euphausiids and chaetognaths. Nevertheless, studies of Auel et al. [24] and Kohlbach et al. [8] using lipid, fatty acid composition and stable isotopes, found evidence for a niche segregation between *T. libellula* and *T. abyssorum* in the Arctic ecosystem, with *T. libellula* being more dependent on the ice-algal pathway, feeding on herbivorous copepods, whereas *T. abyssorum* occupies a higher trophic position, feeding on omni- and carnivorous prey. If these two sympatric species systematically occupy distinct trophic positions and display divergent feeding habits, we can assume that the two amphipods will be differently impacted by the consequences of climate change and sea-ice decline in the Arctic [23].

In order to make reliable predictions on the future Arctic ecosystem, it is crucial to comprehensively characterize the ecological niches and prey spectra of these species in a higher resolution. The majority of previous studies characterizing *Themisto*’s diet are based on stereomicroscopy or biomarker analyses [8,24,26,27]. Morphological analyses are, however, strongly limited in taxonomic resolution and are more efficient in detecting hard-bodied than soft-bodied prey, the latter being often overlooked due to their rapid digestion [28]. The application of biomarkers, such as fatty acids, aims at determining the source of primary production, but also offers limited taxonomic resolution [24]. Molecular analyses provide several advantages over biomarker or morphological analyses, in that they allow us to characterize the prey spectrum in unprecedented resolution, including usually overlooked soft-bodied prey (e.g., pteropods, cnidarians, ctenophores, chaetognaths). As an example, Olsen et al. [27] used denaturing high-performance liquid chromatography with optimized 18S rDNA primers for characterizing *T. abyssorum*’s diet, revealing the presence of cnidarians in some of their guts.

Therefore, we use molecular methods to comprehensively characterize the diet of the two co-occurring *Themisto* species in the Fram Strait region, the rapidly changing gateway to the Arctic Ocean. In this study, we compare the prey spectra between and within the two species, and for each species, we assess its regional variation in diet. We also aim to characterize the role of potentially overlooked soft-bodied zooplankton in the prey spectra of *Themisto* amphipods. We apply DNA metabarcoding of the mitochondrial marker cytochrome *c* oxidase I (COI) on stomach contents, with the purpose of identifying the prey spectra in unprecedented high resolution [29,30]. The outcome of this comprehensive diet study will ultimately allow better predictions on how these key zooplankton species will be impacted by the ongoing climate change and the consequences this will have for the entire Arctic food web.

## 2. Materials and Methods

*Themisto* samples were collected at several locations in Fram Strait between 2016 and 2017 during two R/V Polarstern [31] cruises and the Norwegian TUNU-VII cruise with R/V Helmer Hansen (Table 1, Figure 1). During the R/V Polarstern [31] cruises, zooplankton was collected either using a Bongo net or a Multinet [32,33]. At TUNU stations, samples were collected using a Harstad pelagic trawl or a Campelan bottom trawl. *Themisto* specimens were stored in 96–100% ethanol after identification, that was replaced after ca. 24 h of preservation and kept at −20 °C. Before stomach dissection, the amphipods were roughly measured to the closest 5 mm and attributed to different size classes (<10 mm, 10–20 mm and >20 mm).

DNA from the stomach contents of the amphipods was extracted using the DNeasy PowerSoil kit [34,35], following the instructions of the manufacturer, with a final elution volume of 75 µl To increase the amount of DNA in the samples and the number of sequenced individuals, where possible, up to 10 stomachs were pooled for the larger *T. libellula* (>10 mm) whereas for the much smaller *T. abyssorum* (<10 mm) up to 25 stomachs were pooled [35]. Individuals of the same size class were selected for pooling. From each location three samples were taken each including multiple stomachs, in total that resulted in 18 samples for each *Themisto* species. Stomach contents were removed using dissection tweezers. A lateral incision was made on the pereon, thereafter the pleon was removed, and the gut contents were scraped out. In order to avoid cross-contamination between samples, dissection tools were thoroughly flame-sterilized between processing of different individuals using 70% ethanol. The amount of DNA in the samples was checked immediately after the extractions using a NanoDrop ND-1000 spectrophotometer (Thermo Fisher Scientific, Waltham, MA, USA), before storing the DNA extracts at −20 °C.

The library preparation and sequencing were performed at AllGenetics & Biology SL in Spain (www.allgenetics.eu (accessed on 15 March 2021)). For the library preparation, a two PCR approach was used targeting a 313 bp long fragment of the COI gene called the Leray-fragment [36]. For amplification, a highly degenerated version of the Leray primers was used, with mlCOIintF-XT as the forward primer (5′-GGWACWRGWTGRACWITITAYCCYCC-3’) [37] and jgHCO2198 as the reverse primer (5′-TAIACYTCIGGRTGICCRAARAAYCA-3’) [38]. These primers have been successfully applied for characterizing a diverse set of metazoan prey in diet studies of coral reef fish, northern shrimp (*Pandalus borealis*) and brown shrimp (*Crangon crangon*) [30,35,36]. The first PCR was carried out in a total volume of 25 µL, containing 2.5 µL of DNA, 0.5 µM of Leray-XT primers, 12.5 µL of Supreme NZYTaq 2x Green Master Mix (NZYTech, Lisbon, Portugal), and ultrapure water. The PCR was started with an initial denaturing step at 95 °C for 5 min, before 35 PCR cycles started with denaturing at 95 °C for 30 s, followed by an annealing step at 54 °C for 45 s and an extension at 70 °C for 30 s. In the end, a final extension step at 72 °C for 10 min was applied. During the first PCR, three PCR replicates were created and kept separate throughout the library preparation and sequencing. In a second PCR, the ligation of indices for the multiplexing of samples was performed. AllGenetics & Biology SL used a total volume of 25 µL that contained 2.5 µL of the first PCR product, 0.5 µM indexed primers, 12.5 µL Supreme NZYTaq 2× Green Master Mix (NZYTech) and ultrapure water. The temperature profile for the second PCR was slightly changed to only 5 cycles with an annealing temperature of 60 °C for 45 s. In total, 108 libraries were sequenced. Libraries were first purified using the Mag-Bind RXNPure Plus magnetic beads (Omega Biotek, Norcross, GA, USA) following the instructions of the manufacturer. Quantification was performed with the Qubit High-Sensitivity dsDNA Assay kit (Thermo Fisher Scientific). The libraries were equimolarly pooled and sequenced in a MiSeq PE 2 × 300 run (Illumina, San Diego, CA, USA).

**Figure A1:**
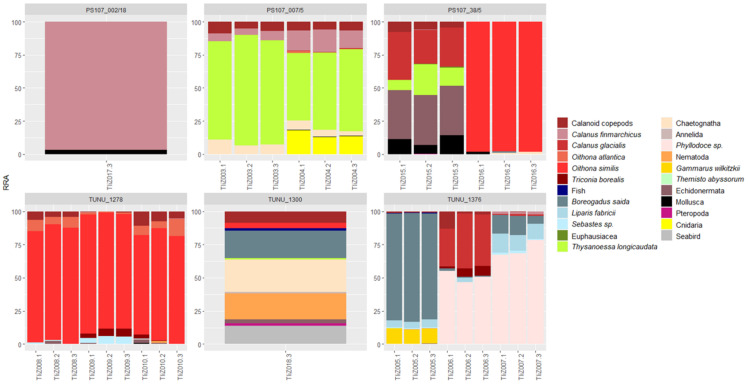
Prey spectrum found in the stomachs of *T. libellula* at different stations, including the PCR replicates per station. Number of reads are given in relative read abundances (RA); colors indicate different prey items; names of x-axis indicate sample IDs. Prey items contributing less than 1%.

**Figure A2:**
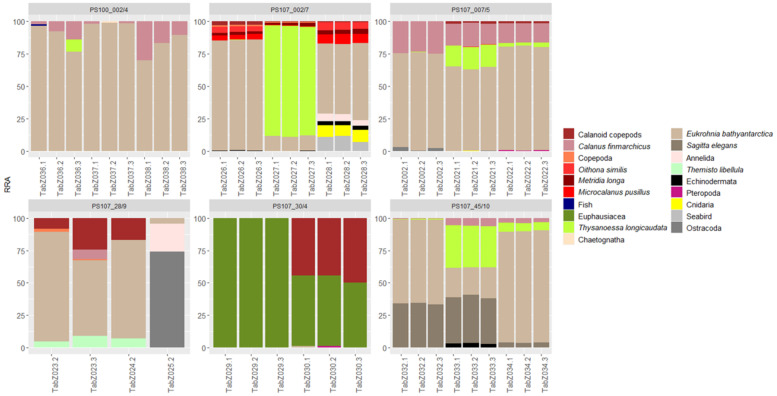
Prey spectrum found in the stomachs of *T. abyssorum* at different stations, including the PCR replicates per station. Number of reads are given in relative read abundances (RA); colors indicate different prey items; names of x-axis indicate sample IDs. Prey items contributing less than 1%.

Quality control of the reads was performed on the forward and reverse reads separately using FastQC [39]. The reads were demultiplexed and sequencing primers and adapters were removed by the sequencing company. Read assembling was done using vsearch (version: v2.15.0_linux_x86_64) [40], allowing 5–10 differences and excluding Ns, with a minimum overlap of 200 bp to reach for at least 80% of assembled reads. The Leray-XT primers were removed using cutadapt (version: 2.10. with Python 3.8.5) with linked primers anchored to the 5′ end. Quality trimming was done using vsearch with a quality threshold of 30, after which sequences shorter than 200 bp were removed from the dataset using vsearch. MOTUs clustering was done using SWARM (d = 13) [41] and the taxonomic assignment was done using Ecotag as part of OBItools [42] and the DUFA reference database (https://github.com/uit-metabarcoding/DUFA/tree/master/DUFA_COLR (accessed on 10 November 2021)). After the taxonomic assignment, only MOTUs that were assigned with at least 90% of similarity were considered. Samples with less than 50 prey sequences were also excluded from the dataset. Taxa with more than 0.05% of prey sequences within each sample were considered as prey taxa whereas those with less than 0.05% were excluded. This exclusion resulted in an underrepresentation of some stations in terms of replicates. For instance, this was true for stations PS107_002/18, PS107_007/5, PS107_38/5 and TUNU_1300 for the samples of *T. libellula*. For *T. abyssorum*, fewer samples were excluded. For this species, one replicate was excluded for station PS107_30/4 and two for station PS107_28/9. Additionally, reads assigned to phytoplankton, bacteria, and non-planktonic organisms, such as insects and terrestrial mammals were removed from the data set. Three PCR replicates were analyzed separately throughout the sequencing process. Since no differences in the prey composition were visible between these PCR replicates, the results were merged for each sample. The PCR replicates are shown in Appendix A (Figure A1 and Figure A2). BioSamples are available at the NCBI database and are accessible by accession numbers SAMN29843075—SAMN29843103. Raw sequences are available at NCBI and accessible by accession numbers SRR20700832 - SRR20700755.

All statistical analyses and data visualization were performed using the RStudio software (R version 4.1.0 (18 May 2021)) [43]. For the data visualization and statistical analysis, relative abundances of reads within each sample (RAs) were used to compare prey items between predators and different localities, which has previously been used for pooled samples [35,44]. To determine differences in the prey spectra between the two predators, as well as the spatial variation within each predator species, canonical correspondence analysis (CCA) and an ANOVA as a permutation test for the complete CCA as well as for each axis were performed. The CCAs were created using RStudio and the vegan package (R package version 2.5-7) and were modified using the package goeveg [45,46]. To evaluate the performance of the CCA, an ANOVA with 1000 permutation steps was used to identify the significance of the two axes used in the model.

## 3. Results

After quality control and length trimming, a total of 2,426,745 reads for *T. libellula* and a total of 1,827,582 reads for *T. abyssorum* were obtained, with an average of 44,940 reads per sample for *T. libellula* and 33,844 reads per sample for *T. abyssorum*. The negative control showed a low number of reads of *T. abyssorum* (1 read) and *T. libellula* (21 reads). No clear differences between the PCR replicates for each sample was found (see Appendix A Figure A1 and Figure A2). The hosts, *T. libellula* and *T. abyssorum*, contributed with an average of 98% and 84% of the reads, respectively. For *T. libellula*, that resulted in a minimum of 94 and a maximum of 11201 prey reads. For *T. abyssorum*, the minimum number of prey reads was 154 and the maximum number was 50219 reads.

### 3.1. Interspecific Variation in the Diet Composition of the Two Themisto Species

**Figure 2:**
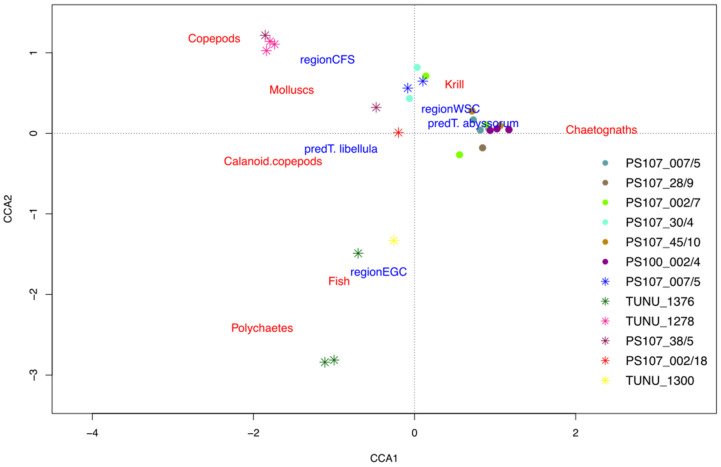
Canonical correspondence analyses **(**CCA) of the diet of the two predators. Colors indicate different stations. Stars represent sampling stations of *T. libellula* and dots indicate sampling stations of *T. abyssorum*. Data is separated by geographical regions and predator species (both as blue text), regions were identified by geographical regions indicated in Figure 1. Only 50% of the most abundant prey species were displayed (red text). The first axis (CCA1) separates the predator species, with *T. libellula* being displayed on the left-hand side and *T. abyssorum* on the right-hand side. The second axis (CCA2) separates the different geographical regions from each other.

**Figure 3:**
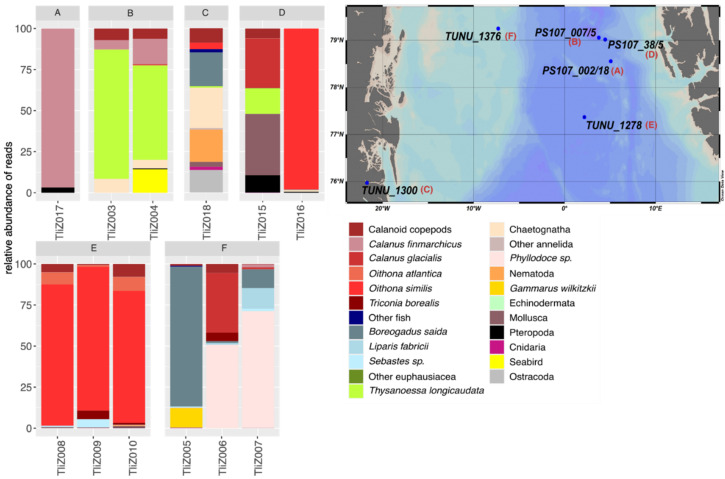
Prey spectrum found in the stomachs of *T. libellula* at different stations. Number of reads are given in relative abundances of reads per sample (RA), colors indicate different prey items, names of x-axis indicate sample IDs. Prey items contributing less than 1% to total prey reads are comprised to phylum level. Copepods are shown in red, fish in blue, and euphausiids shown in green color variants. Stations are represented by letters A–F and are shown in the map.

When comparing the prey spectra between the predator species, we observed differences in terms of major prey groups and regional variability. Overall, *T. libellula*’s diet showed a high variability between different stations, which is evident from the CCA (Figure 2), with different prey species showing high RA values at different stations (Figure 3). The diet of *T. abyssorum* was, in general, more consistent among localities and was often dominated by a single prey species (Figure 2. The diet of *T. libellula* was mainly dominated by copepods, while the prey spectrum of *T. abyssorum* was dominated by chaetognaths (Figure 2). All data points belonging to *T. abyssorum* as predator were accumulating in one area, with chaetognaths and krill being the most important prey species (Figure 2). The data points belonging to *T. libellula* as predator, were widely spread with different prey items being more predominant depending on the sampling area (Figure 2). The CCA explained 32% of the inertia significantly distributed over the two axes CCA1 (16%) and CCA2 (13%). The permutation test showed that both axes were significant. 

### 3.2. Intraspecific and Spatial Variation in Prey Composition of T. libellula

In general, the prey spectrum of *T. libellula* varied between polar stations closer to the Greenland shelf and the warmer Atlantic stations closer to Svalbard (Figure 3). At the polar stations, *T. libellula*’s diet was composed of ice-associated prey such as the sympagic amphipod *Gammarus wilkitzkii* (station TUNU_1376) or of fish species such as *Boreogadus saida* (polar cod), *Sebastes* spp. (redfish) and *Liparis fabricii* (snailfish) (stations TUNU_1300 and TUNU_1376) (Figure 3). At the Atlantic-influenced stations, prey spectra were dominated by boreal-Atlantic copepod species such as *Calanus finmarchicus* or other boreal species such as the krill species *Thysanoessa longicaudata* (Figure 3). At the southernmost station TUNU_1278, *T. libellula*’s diet was dominated by reads assigned to the copepod species *Oithona similis* and *O. atlantica* (Figure 3). At the same station, we also identified a portion of the reads belonging to redfish (*Sebastes* spp.) (Figure 3). The overall pattern observed in the prey spectrum of *T. libellula* showed a shift from a copepod- and krill-dominated diet at Atlantic-influenced stations to a more mixed diet at the polar stations (Figure 3). At most stations, the variation between samples appeared to be high, except for station TUNU_1278, where the three sample replicates were dominated by the same type of prey (Figure 3). At station PS107_38/5, one sample contained almost exclusively reads assigned to *C. finmarchicus*, while the other sample from the same location showed a mixed composition, dominated by reads of *C. glacialis* and mollusks, including the pteropod *Clione limacina* and the bivalve *Hiatella* sp. (Figure 3). Another notable finding was the high RA of the annelid *Phyllodoce* sp. at station TUNU_1376 close to the Greenland shelf, where relatively high RAs of this prey item were detected in two out of three replicates. At station PS107_007/5, the krill species *T. longicaudata* was dominating in one sample but showed lower RA in the other sample. Low RAs of different cnidarian taxa, including the hydrozoans *Catablema vesicarium*, *Aglantha digitale* and *Obelia longissima*, as well as the siphonophore species *Nanomia cara* and *Rudjakovia plicata*, were occasionally observed, with a particularly pronounced signal at station TUNU_1300. Here, higher RAs of the siphonophores *Nanomia cara* and *Rudjakovia plicata* were observed. In the second sample of this station, reads belonging to the seabird *Alle alle* (little auk) were detected.

**Figure 4:**
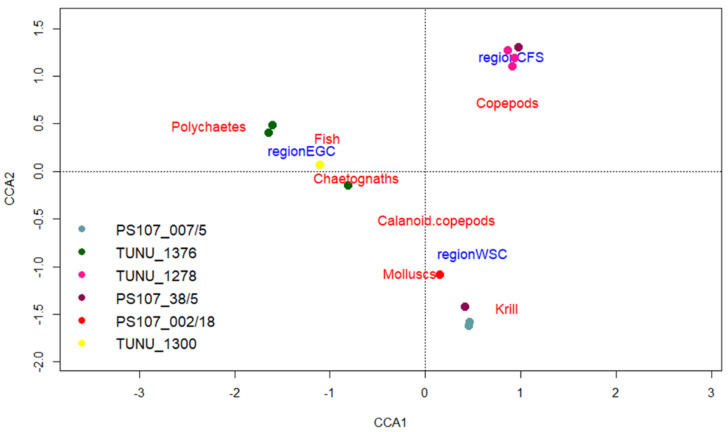
Canonical correspondence analyses (CCA) of the spatial variation of prey items for *T. libellula*. Colors indicate different stations; regions are identified by geographical region indicated in Figure 1. Only 50% of the most abundant prey species are displayed. The first axis (CCA1) separates the EGC region from the other two regions, with the EGC region being located on the left-hand side. The second axis (CCA2) separates the two Atlantic regions from each other, with the CFS regions in the upper part and the WSC regions in the lower part.

Additionally, the intraspecific and spatial variance in the prey spectrum of *T. libellula* was investigated using CCA (Figure 4). The permutation test showed that the CCA significantly explained 34% of the total inertia (*p*-value: 0.006). This was mainly attributed to the first axis with 20% significance (*p*-value: 0.008), while the second axis was not significant, but still explained 13% of the variation. The CCA showed that the prey spectrum at stations located on the Greenland shelf was mostly dominated by polychaetes and fish species, while at stations close to the Svalbard shelf, krill and calanoid copepods dominated the diet. At the stations in central Fram Strait, *T. libellula*’s diet was dominated by copepods (Figure 4).

### 3.3. Intraspecific and Spatial Variation in Prey Composition of T. abyssorum

**Figure 5:**
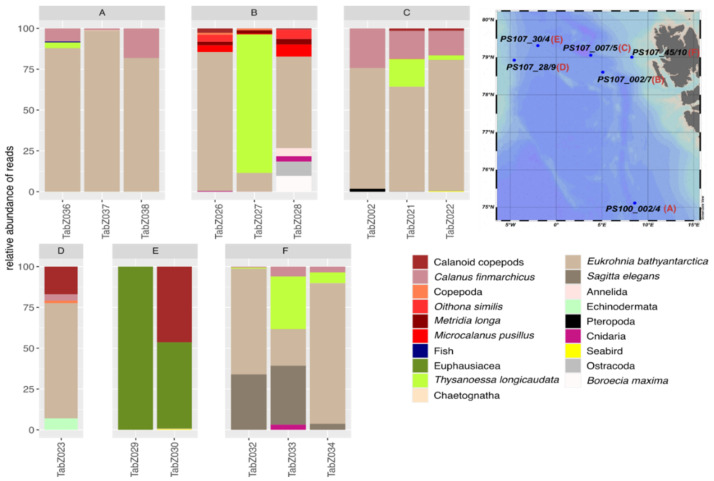
Prey spectrum found in the stomachs of *T. abyssorum* at different stations. Number of reads are given in relative abundances of reads per sample (RA); colors indicate different prey items; names of x-axis indicate sample IDs. Prey items contributing less than 1% to the total prey reads are comprised to phylum level. Copepods shown in red, euphausiids shown in green and chaetognaths shown in beige and brown color variants. Stations are represented by letters A–F and are shown in the map.

Overall, only small variations within the diet composition of *T. abyssorum* were found among the different locations (Figure 5). The same was true for the variation within stations, where prey spectra did not vary as strongly as they did for *T. libellula*. *T. abyssorum’s* diet was dominated by chaeatognaths: the highest RAs were represented by the chaetognath *Eukrohnia bathyantarctica*, followed, at some stations, by the chaetognath *Sagitta elegans* as the second dominant species (Figure 5). Only at station PS107_30/4, were no reads of chaetognaths identified. At this station, the prey composition of *T. abyssorum* was dominated by euphausiids. The krill species *Thysanoessa longicaudata* was also dominant in *T. abyssorum*’s diet at other locations (PS107_00/4, PS107_45/10 and PS107_007/5) (Figure 5). In general, copepod reads were less dominant, and were represented by different copepod species such as *C. finmarchicus* and *Metridia longa* (Figure 5). At station PS107_28/9, the chaetognath *E. bathyantarctica* was represented with high RAs in two of the replicates, whereas pteropods had the highest RA in the third replicate (Figure 5). In the two other samples, besides chaetognath and copepod reads, sequences assigned to echinoderms were found. Although copepods never comprised the largest portion of reads in the samples, a high diversity of copepods and ostracods was found, including *M. longa*, *Boroecia maxima*, *Oithona similis* and *Microcalanus pusillus*, nevertheless calanoid copepods were making up the largest fraction of copepod reads (Figure 5). At stations PS107_002/7 and PS107_45/10, cnidarian reads were identified in two samples (Figure 5). These made up only a small portion of the overall reads but were more abundant at these stations compared to others. At station PS107_007/5, we found reads assigned to the seabird *A. alle*, which was at the same station where we also found this species in *T. libellula*’s stomach content DNA, albeit in higher RAs than seen here for *T. abyssorum* (Figure 5).

**Figure 6:**
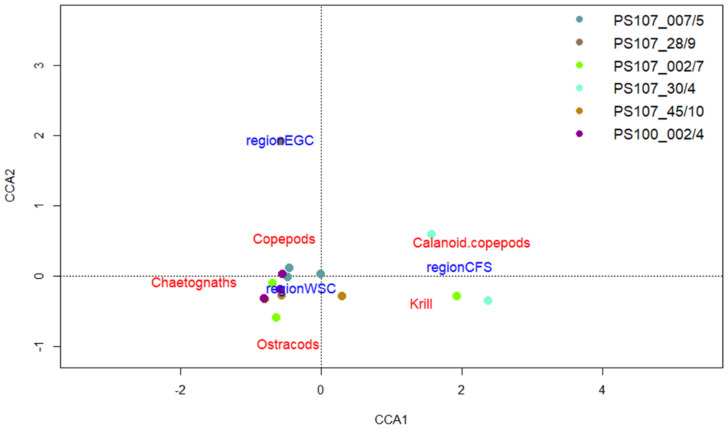
Canonical correspondence analyses (CCA) of the spatial variation of prey items for *T. abyssorum*. Colors indicate different stations; regions are identified by geographical region indicated in Figure 1. Only 50% of the most abundant prey species are displayed. The first axis (CCA1) separates the two Atlantic regions from each other, with the WSC region being located on the left-hand side. The second axis (CCA2) separates the EGC region from the other two geographical regions, with the EGC regions in the upper part.

As already seen in the prey composition of *T. abyssorum* (Figure 5), the variance among stations was less obvious compared to *T. libellula*, also demonstrated by the CCA results for *T. abyssorum* (Figure 6). The permutation test showed that the full model was significant (*p*-value: 0.017), but only the first axis showed a significance (*p*-value: 0.01). In total, the model explained 37% of the inertia significantly, with 30% explained by the first axis and 6% by the second axis. Nevertheless, the model showed chaetognaths contributed most to the prey spectrum at most stations, while only few stations were dominated by calanoid copepods or krill. 

## 4. Discussion

### 4.1. Interspecific Variation in the Diet Composition of the Two Themisto Species

Using DNA metabarcoding, we provided the first high resolution prey spectra of two *Themisto* amphipods in the Arctic region. The results revealed a highly divergent diet of the two *Themisto* species, among the sampled locations in the summer season (July–September). Dalpadado et al. [47] showed, based on light microscopy, that *T. libellula* mainly feeds on calanoid copepods, whereas *T. abyssorum* had a broader prey spectrum including copepod species, but also other zooplankton such as appendicularians. Using lipid analyses, Auel and colleagues [24] found that *T. libellula* is more dependent on sea-ice algal primary production, whereas *T. abyssorum* shows a higher degree of carnivory, feeding on omnivorous or carnivorous prey. With DNA metabarcoding as a novel method in the field of diet studies, we corroborated that the prey composition of both species generally strongly differed, but we were also able to reveal their prey diversity at an unprecedented high taxonomic resolution. In the diet of *T. libellula*, high read numbers of copepod species were detected at the Atlantic influenced stations (*Calanus finmarchicus* and *Oithona similis*), where also the krill species *Thysanoessa longicaudata*, pteropods (*Clione limacina*) and bivalves (*Hiatella* spp.) were found in relatively high read numbers. At Arctic-influenced stations (sampled in September), copepods seemed to be less important as prey. Here, reads belonging to fish species such as *Boreogadus saida* and *Sebastes* spp. or annelids (e.g., *Phyllodoce* sp.) were identified. These results indicate that *T. libellula* feeds on a broad prey spectrum, which was not found in other studies using light microscopy targeting the Barents Sea and Svalbard fjords [47,48]. For *T. abyssorum*, previous studies suggested a broad prey spectrum in summer months [24,47], whereas in winter months the diet was exclusively composed of copepod species [48]. Our results show a dominance of chaetognaths such as *Eukrohnia bathyantarctica* and *Sagitta elegans* in the diet of *T. abyssorum*, and to a lesser extent, euphausiids, echinoderms and mollusks (pteropods). This is in clear contrast to the prey found for *T. libellula*, for which almost no chaetognath reads were recorded. Although a higher diversity of prey organisms was identified in our study, the overall pattern here shows a different diet composition between the two predators during the summer and autumn months This is in alignment with results from other studies, showing an interspecific difference in prey spectra, with only a partial overlap in the predation of calanoid copepods [24,47]. In our study, we found only a fraction—depending on the region—being assigned to calanoid copepods. This lesser dominance of copepods in our molecular study can be explained by the fact that calanoid copepod parts are easily identified with microscopic stomach analyses, whereas other prey remnants are more easily overlooked or more challenging to assign morphologically. Contrary to the interspecific variation found for summer diets, Kraft and colleagues [48] found no difference in winter diet composition between the species sampled in the Svalbard fjords, based on light microscopy. Here, *C. finmarchicus* CV copepodites were found to be the dominant prey [48].

A study by Dalpadado and colleagues [49], investigated the distribution of Arctic *Themisto* species and showed that *T. libellula* and *T. abyssorum* segregate at different depths. This depth segregation could explain the difference in diet composition observed herein. However, depth segregation may be more tenuous as previously assumed [23], as swarms of both species have been observed near the seafloor in deep-sea regions [50,51], and *T. abyssorum* individuals have been observed feeding on detritus on the seafloor (800–1270 m depth) [50]. We found DNA of organisms with a benthic adult stage (*Phyllodoce* polychaetes and bivalves); however, we cannot identify whether this originates from the predation on meroplanktonic larvae or on benthic adults. We identified the deep-sea chaetognath *Eukrohnia bathyantarctica* as part of the diet of *T. abyssorum*. *E. bathyantarctica* is mostly found in deeper waters of 1500–2000 m and also newly hatched juveniles are found at depths below 1000 m [52]. Nevertheless, Kruse and colleagues [52] observed the newly hatched chaetognaths (6 mm length) in depths of 500–750 m during the summer time. Since we cannot distinguish between the developmental stages of the ingested *E. bathyantarctica* it remains uncertain if the observed chaetognath predation is an indication of deep migration. Deep migrations of *Themisto* would have major implications for pelagic-benthic coupling processes and the biological carbon pump [23], hence, this should be further investigated with combined metabarcoding and biomarker studies.

The size difference of the investigated specimens for each species is another variable to consider for explaining the differences in the dietary composition. A change in feeding habits from *Themisto* juveniles to adults has been reported using light microscopy and biomarkers, with a switch from a more herbivorous to a carnivorous diet [23]. The specimens of *T. abyssorum* investigated here were mostly smaller than 10 mm, corresponding to juveniles or immature adults. We did not include the non-metazoan taxa recovered by the COI primer set, as i) this primer set is most commonly validated to identify metazoans [37], and ii) it is impossible to reliably distinguish between grazing from the ingestion of phytoplankton through secondary predation. With our results, we could demonstrate carnivory in all juvenile *T. abyssorum* analyzed. For *T. libellula*, we sampled larger specimens >10 mm, which were most likely mature adults. The two *Themisto* species can reach different maximum sizes, and the differences between analyzed specimens of the two species is a likely reason for the observed differences in the prey spectra. Overall, we were able to show the advantages of our methodology. Further DNA metabarcoding studies can be applied to compare the diet of different life stages and size classes of *Themisto* targeting different gene fragments to determine a potential change in feeding habits.

We were able to identify a broad range of prey organisms for both species, which were not reported in previous studies based on light microscopy or biomarkers. For example, we detected predation on gelatinous zooplankton and other soft-bodied organisms, as well as feeding on different fish species. Gelatinous zooplankton predation has been reported in previous studies for *T. abyssorum* [27,50], but not for *T. libellula*. Since the resolution and accuracy of the taxonomic assignment is strongly dependent on the quality of the reference database, COI might not be the most adequate marker for the detection of particular taxa. For different gelatinous organisms, such as appendicularians, ctenophores and hydrozoans [53,54,55], COI primers do not always amplify all taxa, and existing reference databases are more complete for other gene fragments than COI (18S rDNA or 16S rDNA). Nevertheless, we showed that gelatinous zooplankton is a factor in the diet of *Themisto* and it seems to be even more significant in terms of interspecific variation. To further investigate the role of gelatinous zooplankton, it is advised to include additional target genes, such as 18S rDNA or 16S rDNA, which are shown to amplify gelatinous taxa more reliably, however, with a lower taxonomic resolution [37,56,57]. 

### 4.2. Intraspecific and Spatial Variation in Prey Composition of T. libellula

To our knowledge, this is the first DNA metabarcoding diet study on *T. libellula*, hence, this allowed us to detect hitherto overlooked prey species. Our results showed that *T. libellula* is able to feed on a variety of prey items, from copepods to krill, as well as, amongst others, fish, gelatinous zooplankton, and annelids, which have not yet been reported for this species. Other studies using light microscopy or biomarkers concluded that *T. libellula* has a narrow prey spectrum dominated by calanoid copepods [24,47]. We showed that calanoid copepods indeed make up a large proportion of the diet during the summer months in Fram Strait, but other species such as *Thysanoessa longicaudata* and *Oithona similis* can also be dominant prey taxa. Since *T. libellula* is known to be a cryptic species complex [58], the broad prey spectrum observed herein could be a result of different cryptic species each potentially having different or more restricted diets. This point should be investigated in future studies. At some stations, several prey taxa appeared in similar proportions. This was the case for station TUNU_1300, where *Boreogadus saida*, nematodes, copepods and chaetognaths were present in similar RA. The dominance of one taxon in some samples and the presence of several taxa in equal proportions in other samples, can be explained by the ingestion of one big prey item in one case and the ingestion of several smaller parts in the other. Such an overdominance of one prey item in terms of reads is more likely to happen in diet studies investigating the stomach content of every individual separately; pooling several individuals can decrease the likelihood of such a bias, however, not fully exclude it. Fish species such as *B. saida* or *Sebastes* spp. could also be detected at some locations, which could reflect feeding on fish eggs, larvae, feces or scavenging behavior. Predation on fish larvae has so far not been reported for *T. libellula*; here, fish DNA was only detected at western stations close to Greenland in September. Williamson [59] found evidence for the predation on “post-larval” fish by *T. compressa* (previously *T. gracilipis*).

Apart from these new insights revealing so far unreported prey in the diet of *T. libellula*, we also demonstrated spatial variation in its diet in Fram Strait. The diet composition in the summer season appeared to be spatially structured, with the prey composition based on RAs and the CCA model as showing distinct patterns between the different locations. The results indicated a tendency towards a copepod- and krill-dominated diet in Atlantic influenced stations (WSC) and fish predation and ice-associated prey at polar stations (EGC). Thus, we assume that this local variation in feeding of *T. libellula* is determined by changes in the prey field due to the local environmental conditions. In previous lipid biomarker studies, the role of sympagic production was shown to be important for *T. libellula* [24]. Under the locally and temporally variable influence of sea-ice in the investigated area, we showed a spatial variability in *T. libellula*’s diet, which may indicate a flexibility to switch to a diet dominated by Atlantic-boreal species. This flexibility could be important in the context of decreasing sea-ice and warming of the Arctic Ocean.

In line with previous diet studies on *T. libellula*, we were able to detect predation on calanoid copepods, but additionally, we found a high prey diversity encompassing different taxa, including fish species and mollusks, especially pteropods. A species that was not particularly reported as prey in previous studies is the polychaete *Phyllodoce* sp., found in high RAs at the northernmost station close to the Greenland shelf. It is known that this polychaete is widely distributed, including the shelf regions and fjords in east Greenland [60,61,62]. As adults have a benthic mode of life, it is likely that the predation on this species happens through feeding on meroplanktonic larvae. Spawning of *P. groenlandica* happens during spring and early summer in the Kara Sea [60]. Fetzer and Deubel [60] reported a relatively long pelagic stage for the trochophores, and the larvae were reported to remain in the water column until September; so we assume that *T. libelulla* was able to feed on its planktonic larvae. Finally, DNA assigned to the little auk (*Alle alle*) was found in the diets of *T. libellula* and *T. abyssorum* at the same station; thus, we assume that the two *Themisto* either scavenged or fed on scat, or the presence of DNA is a result of feeding on feathers or scat in the cod-ends of the nets.

### 4.3. Intraspecific and Spatial Variation in Prey Composition of T. abyssorum

No spatial structure was observed in the prey spectrum of *T. abyssorum*, which was supported by the non-significant CCA. Most of the samples had a similar prey composition with chaetognaths being the most important prey species. Previous studies suggested a broad prey spectrum and a high level of carnivory for *T. abyssorum* [24,47]. We showed a diversity of prey DNA in *T. abyssorum*’s stomachs, confirming that it feeds on a wide range of zooplankton species, but the diet was dominated by the chaetognath *Eukrohnia bathyantarctica* at most locations. Copepods seemed to play a minor role in the diet of this *T. abyssorum*. This was rather surprising, considering the size of the specimens (<10 mm) included in the analysis, suggesting that they were juveniles or immature adults. Other studies suggested a diet dominated by phytoplankton for juvenile *Themisto* [23]. As mentioned before, phytoplankton was removed from this analysis due to marker choice and possible secondary predation. Nevertheless, we were able to identify a high level of carnivory even for juvenile *T. abyssorum*. Unlike for *T. libellula*, *T. abyssorum*’s dietary composition has already been investigated with molecular tools. Olsen et al. [27] used a denaturing high-performance liquid chromatography with a universal 18S rDNA primer set to assess the diet of *T. abyssorum* at three hydrothermal vents in the Arctic. They found that hyperiids make up a large proportion of the diet of *T. abyssorum* [27]. In our samples collected in Fram Strait, we did not find any reads assigned to hyperiid species (except those assigned to the target species *T. abyssorum* and *T. libellula*). Other than hyperiids, Olsen and colleagues [27] identified calanoid copepod species made up larger parts of the prey spectrum at some locations, whereas in our study they only seemed to play a minor role, suggesting that *T. abyssorum* may utilize different prey depending on the available prey field. However, we were able to identify a variety of other copepod species, with *Oithona similis* and *O. atlantica* dominating at the southernmost station. The predominance of the chaetognath *E. bathyantarctica* in most stations can be explained by their high abundance during summer in the Arctic Ocean. Considering the size of the investigated *T. abyssorum* in this study, we assume that it fed on eggs or newly hatched larvae of *E. bathyantarctica* and *S. elegans*. At stations with low RAs of chaetognaths, *T. abyssorum*’s diet was rather dominated by Euphausiacea, such as *T. longicaudata*.

### 4.4. The Trophic Role of Themisto in a Changing Arctic Ocean

Since the Arctic is highly affected by climate change, reflected in an increased “Atlantification” and sea-ice loss, it is urgent to gain an in-depth baseline knowledge of the diet spectra of the *Themisto* amphipods allowing for accurate predictions of climate-induced trophic shifts. *T. libellula* is a genuine polar, cold-water species, of which populations are decreasing [25], and likely to be severely impacted by increasing seawater temperatures. In our study, we demonstrated a spatial heterogeneity in its diet spectrum, indicating trophic flexibility and opportunistic feeding behavior. This spatial variance could also indicate locally adapted populations utilizing different food sources. Further investigations should be carried out on a broader geographic scale looking into the population genetics combined with feeding preference. Ice-associated taxa made up an important part of *T. libellula*’s diet in cold polar waters near the East Greenland shelf, whereas boreal-Atlantic prey, such as *Calanus finmarchicus*, dominated in Atlantic-influenced localities near the Svalbard shelf. *C. finmarchicus* is anticipated to replace the Arctic copepod *Calanus glacialis* in a more atlantified Arctic [63,64]; hence, *T. libellula* may be able to utilize this prey as it will become an increasingly abundant species in the Arctic. Other prey species, detected in the stomach contents of *T. libellula*, will be negatively impacted by warming and sea-ice retreat, including polar cod *Boreogadus saida* and polar copepod species [8,11,12,65,66]. It remains unclear how such shifts in the zooplankton composition in the future Arctic will impact the feeding conditions for both species. *T. libellula* may not only be affected by abiotic changes, but also by the loss of important prey species. However, its broad prey spectrum might enable *T. libellula* to feed on the increasingly abundant Atlantic species, but it remains unknown how this shift to less lipid-rich boreal-Atlantic prey species will eventually affect its fitness. For *T. abyssorum*, an expansion of its distribution range has been predicted [23]; however, its prey spectrum appears to be less variable, potentially indicating a lesser flexibility to cope with changes in the available prey field. As *Themisto* amphipods are major predators throughout the Arctic Ocean, it is pivotal to gain a better understanding of the spatio-temporal variation in their prey spectra. A combination of biomarker and metabarcoding studies are needed to obtain a more complete picture of *Themisto*’s diet, as well as prey preference studies investigating, also, the available prey field. Such data are of crucial importance to accurately predict the fate of these key species in the Arctic food web and the consequences of potential trophic shifts for Arctic top consumers, such as seabirds, fish, and marine mammals.

## Figures and Tables

**Table 1:** Overview of stations and sampling methods during PS100, PS107 and TUNU-VII. BO = Bongo net, PT = Pelagic trawl, CT = Campelan bottom trawl, MN = Multi net. Depth refers to sampling depth at the stations.

Station	Species	Size [mm]	Latitude [°N]	Longitude	Gear	Depth [m]	Date
PS107_007/5	*T. libellula*	10–20	79.06	3.75 E	BO	450	29 July 2017
TUNU_1376		10–20	79.25	7.31 W	PT	20	24 September 2017
TUNU_1278		10–20	77.37	2.15 E	PT	31	15 September 2017
TUNU_1300		>20	75.97	21.72 W	CT	230	18 September 2017
PS107_002/18		10–20	78.56	5.06 E	BO	60	26 July 2017
PS107_38/5		10–20	79.02	4.45 E	BO	60	11 August 2017
PS107_007/5	*T. abyssorum*	<10	79.06	3.75 E	BO	450	29 July 2017
PS107_28/9		< 10	78.93	4.58 W	BO	450	5 August 2017
PS107_002/7		<10	78.61	5.06 E	MN	NA	26 July 2017
PS107_30/4		10–20	79.32	2.01 W	BO	450	6 August 2017
PS107_45/10		<10	79.01	8.23 E	BO	450	14 August 2017
PS100_002/4		<10	75.11	8.54 E	BO	NA	20 July 2016

## Data Availability

Raw sequencing data was submitted to SRA and is accessible via the BioProject accession number PRJNA860358. The data is also available through the following link https://www.ncbi.nlm.nih.gov/sra/PRJNA860358.
